# Genomic Rearrangements and Sequence Evolution across Brown Algal Organelles

**DOI:** 10.1093/gbe/evab124

**Published:** 2021-06-01

**Authors:** Samuel Starko, Trevor T Bringloe, Marybel Soto Gomez, Hayley Darby, Sean W Graham, Patrick T Martone

**Affiliations:** 1 Department of Biology, University of Victoria, Victoria, Canada; 2 Department of Botany & Biodiversity Research Centre, University of British Columbia, Vancouver, Canada; 3 Department of BioSciences, University of Melbourne, Melbourne, Australia

**Keywords:** genome colinearity, genome complexity, horizontal gene transfer, kelp, Laminariales, organellar intron, plastid genome, sequence evolution

## Abstract

Organellar genomes serve as useful models for genome evolution and contain some of the most widely used phylogenetic markers, but they are poorly characterized in many lineages. Here, we report 20 novel mitochondrial genomes and 16 novel plastid genomes from the brown algae. We focused our efforts on the orders Chordales and Laminariales but also provide the first plastid genomes (plastomes) from Desmarestiales and Sphacelariales, the first mitochondrial genome (mitome) from Ralfsiales and a nearly complete mitome from Sphacelariales. We then compared gene content, sequence evolution rates, shifts in genome structural arrangements, and intron distributions across lineages. We confirm that gene content is largely conserved in both organellar genomes across the brown algal tree of life, with few cases of gene gain or loss. We further show that substitution rates are generally lower in plastid than mitochondrial genes, but plastomes are more variable in gene arrangement, as mitomes tend to be colinear even among distantly related lineages (with exceptions). Patterns of intron distribution across organellar genomes are complex. In particular, the mitomes of several laminarialean species possess group II introns that have T7-like ORFs, found previously only in mitochondrial genomes of *Pylaiella* spp. (Ectocarpales). The distribution of these mitochondrial introns is inconsistent with vertical transmission and likely reflects invasion by horizontal gene transfer between lineages. In the most extreme case, the mitome of *Hedophyllum nigripes* is ∼40% larger than the mitomes of close relatives because of these introns. Our results provide substantial insight into organellar evolution across the brown algae.

Significance

Organellar genomes are important models for understanding genome evolution across the tree of life. However, both plastid and mitochondrial genomes are poorly characterized in many lineages. We sequenced 20 novel mitochondrial genomes and 16 novel plastid genomes from brown algae (Phaeophyceae) to explore rates of sequence evolution, patterns of structural reorganization, and overall gene content. Our results provide a robust view of organellar genome evolution in an ecologically and economically important taxonomic group.

## Introduction

Plastids and mitochondria are eukaryotic organelles that have evolved after ancient endosymbioses (Margulis 1970; Keeling 2004; Smith and Keeling 2015) and typically retain their own genomes. Organellar genes and genomes have been used as essential phylogenetic markers, both to distinguish species (e.g., Ratnasingham and Hebert 2007; Graf et al. 2017; Hind et al. 2019) and to reconstruct recent and deep evolutionary relationships (e.g., Rai et al. 2003; Janouškovec et al. 2013). Organellar genomes have variable sequence evolution rates across taxa, between organelles (i.e., plastid vs. mitochondrion), and across genes within a single genome (Smith and Keeling 2015). The evolutionary rates of base substitutions and genome structural reconfigurations in both the organellar genomes have long been a focus of research because of their utility in reconstructing and understanding broad patterns in genome evolution (Clegg et al. 1994; Olmstead and Palmer 1994; Smith and Keeling 2015). Despite this, the organellar genomes of many taxonomic groups remain poorly characterized, limiting our ability to draw generalizations about the evolution of organellar genome diversity across the eukaryotic tree of life.

Brown algae (Phaeophyceae, Ochrophyta) are a clade of multicellular algae (i.e., macroalgae) that have tremendous ecological and economic importance globally (Steneck et al. 2002; Bennett et al. 2016; Mac Monagail et al. 2017; Teagle et al. 2017; Wernberg et al. 2019; Bringloe et al. 2020). Large brown algae (e.g., Laminariales) form underwater forests that provide habitat for a wide range of animals and form the base of coastal food webs that fuel the growth of higher trophic levels (Duggins et al. 1989; Steneck et al. 2002; Teagle et al. 2017). The photosynthetic function of brown algae and many other stramenopiles depends on their golden-brown plastid, acquired from an ancient secondary endosymbiosis event of a red alga (Keeling 2004; Sanchez‐Puerta and Delwiche 2008) that occurred sometime prior to the last common ancestor of the photosynthetic stramenopiles. Consequently, the brown algae provide a case study into the evolution of red algal-derived plastids following hundreds of millions of years of evolution since the secondary endosymbiosis event. Despite this, relatively few full brown algal organelles have been sequenced and assembled to date (Bringloe et al. 2020). This limits our ability to recognize and interpret patterns of organellar genome evolution across the group. Moreover, studies on the rates of sequence evolution, patterns of structural evolution, and the prevalence of introns across brown algal organelles have been mostly uniorganellar (e.g., Liu et al. 2015, 2019; Zhang et al. 2015; Yang et al. 2016; but see Graf et al. 2017). Thus, patterns of evolution across plastids and mitochondria have yet to be thoroughly compared in parallel across different brown algal taxa, further limiting our understanding of organellar genome evolution across this globally significant group.

The distribution of introns in the brown algae has also been challenging to determine from current sampling. For example, it is thought that a group I intron in the plastid tRNA-Leu locus of multiple brown algae was likely present in the cyanobacterial endosymbiont involved in the primary endosymbiosis event, as it is widely distributed across the eukaryotic tree of life (Kuhsel et al. 1990; Simon et al. 2003). However, this intron has been found to have an inconsistent distribution both within and across brown algal species (e.g., Corguillé et al. 2009; Zhang et al. 2015), suggesting that intron loss may be a common process across them. Moreover, introns in the mitochondrial genomes of brown algae have only been documented from a single species, *Pylaiella littoralis* (Ectocarpales), but in multiple genes in this genome. Thus, it has been hypothesized that these mitochondrial introns are due to recent, recurrent invasions (Ikuta et al. 2008), an inference based on a very limited sampling of brown algal organellar genomes.

Here, we describe several newly sequenced mitochondrial and plastid genomes of class Phaeophyceae to document and explore organellar genome evolution more comprehensively across the brown algae. To date, most of the published organellar genomes of Stramenopiles are outside brown algae (i.e., 147 of 215 organellar genomes as o f April 2021, https://www.ncbi.nlm.nih.gov/genome/organelle) and around half of the published brown algal organellar genomes are from just two genera: *Saccharina* and *Sargassum*. Here, we focus on the ord ers Laminariales and Chordales, but also include representatives of Ralfsiales, Sphacelariales, and Desmarestiales. We compare these and previously published brown algal organellar genomes to investigate how structural and sequence evolution varies across organelles and lineages. Specifically, we ask how plastomes and mitochondrial genomes compare in terms of 1) overall gene content, 2) rates of sequence evolution (both within and across genomes), 3) the extent of structural rearrangements, and 4) variation in the number and distribution of introns.

## Results and Discussion

### Genome Features

We assembled 41 complete organellar genomes de novo: 18 plastid and 23 mitochondrial, of which 16 plastomes and 20 mitomes are the first sequenced of their species. All genomes had at least 149× mean coverage and mapped as complete circles. Additionally, we attempted to assemble the mitochondrial genome of *Protohalopteris* sp. (order Sphacelariales) as a full circle but were unable to do so due to a complex assembly graph (i.e., likely contamination by additional eukaryotic species) and an apparent repeat region that together made assembly using short reads impossible. We assembled and annotated a large fragment of this mitome (in addition to the 41 complete organellar genomes presented), which nonetheless offered information on overall gene content and structure.

Newly sequenced plastomes varied from 128,910 to 131,355 bp and were all AT rich, ranging from 68.53 to 69.72% AT content. Our results indicate that the plastomes of brown algae consist of a core set of 173 unique genes: 141 protein-coding sequences, three unique rRNA genes (that are each duplicated in the inverted repeat region), typically 28 (but up to 31) tRNA genes, along with a widely conserved ORF occurring between *rps*4 and *ycf*65, adding slightly to the gene totals of previous studies (Graf et al. 2017). The inverted repeat regions are typically ∼5,500 bp in length, capturing mostly rRNA genes but occasionally capturing flanking genes *ycf*37 and *rpl*21. However, the repeat region can be greater than 8600 bp, as in *Ectocarpus siliculosus* (Ectocarpales; table 1). Newly sequenced mitomes varied from 37,097 to 52,071 bp and were also all AT rich, ranging from 59.24% to 67.74% AT content. These ranges are in line with other sequenced organellar genomes in Stramenopiles (e.g., Smith 2012). Our results further confirm that mitochondrial genomes of brown algae consist of 35 protein-coding genes, three rRNA genes, typically 25 tRNA genes, and three widely conserved ORFs (as documented by Secq et al. 2002; Li et al. 2015).

**Table 1.** Structural Information for Brown Macroalgal Organelles

Specimen	Order	Genbank	Mitochondrial				Genbank	Plastid			
			Core	tRNAs	ORFs	Size (bp)		Core	tRNAs	ORFs	*Size (bp)*
Previously sequenced											
* Ishege okamurae*	Ishegeales	MG940857	39^a^	22	4	35,485	—	—	—	—	—
* Dictyopteris divaricata*	Dictyotales	NC_043845	38	25	3	32,021	KY433579	145^b^^,c^	28	0	126,099
* Dictyota dichotoma*	Dictyotales	NC_007685	38	25	3	31,617	—	—	—	—	—
* Fucus vesiculosus*	Fucales	NC_007683	38	26	3	36,392	NC_016735	146^d^	28	0	124,986
* Sargassum fusiforme*	Fucales	NC_024655	38	25	2	34,696	NC_048511	146^d^	28	0	124,298
* Desmarestia viridis*	Desmarestiales	NC_007684	38	25	4	39,049	—	—	—	—	—
* Pylaiella littoralis*	Ectocarpales	NC_003055	38	24	17*^†^	58,507	—	—	—	—	—
* Ectocarpus siliculosus*	Ectocarpales	NC_030223	38	25	5	37,189	FP102296	147^e^	31	4^f^	139,954
* Pleurocladia lacustris*	Ectocarpales	NC_032046	38	26	7	37,814	KU164872	147^b^^,g^	30	2^h^	138,844
* Scytosiphon promiscuus*	Ectocarpales	—	—	—	—	—	MK107984	147^b^	28	2	134,358
* Laminaria digitata*	Laminariales	AJ344328	38	25	4^†^	38,007	NC_044689	147	29	1	130,377
* Laminaria hyperborea*	Laminariales	NC_021639	38	25	4^†^	37,976	—	—	—	—	—
Newly sequenced											
* Protohalopteris* sp.	Sphacelariales	MZ156064	33^	24^	5^	41,306^	MZ156028	148^b^	28	2	131,355
* Analipus japonicus*	Ralfsiales	MZ156065	37^i^	25	4	38,173	—	—	—	—	—
* Desmarestia aculeata*	Desmarestiales	MZ156052	38	25	4^†^	40,822	*MZ156041*	147	28	1	129,228
* Akkesiphycus lubricus*	Chordales	MZ156045	38	25	3	39,330	MZ156027	147	28	1	128,910
* Chorda asiatica*	Chordales	MZ156050	38	25	5	41,788	MZ156037	147	28	0	130,274
* Pseudochorda nagaii*	Chordales	MZ156063	38	25	6^†^	40,990	MZ156030	147	29	1	129,340
* Alaria marginata*	Laminariales	MN395660	38	25	3	38,591	MZ156044	147	29	1	130,568
* Arthrothamnus bifidus*	Laminariales	MZ156049	38	25	3	38,790	MZ156043	147	29	1	130,498
* Costaria costata*	Laminariales	—	—	—	—	—	MZ156042	147	28	1	129,931
* Cymathaere triplicata*	Laminariales	MZ156051	38	25	3	37,998	—	—	—	—	—
* Dictyoneurum californicum*	Laminariales	*MZ156053*	38	25	3	37,840	—	—	—	—	—
* Eiseniaarborea*	Laminariales	MZ156048	38	25	4*	39,843	MZ156038	147	29	1	130,965
* Ecklonia radicosa*	Laminariales	MZ156054	38	25	3	37,577	MZ156040	147	29	1	130,860
* Egregia menziesii*	Laminariales	—	—	—	—	—	MZ156039	147	29	1	130,044
* Hedophyllum nigripes* (AK)	Laminariales	MZ156047	38	25	9*^†^	52,041	—	—	—	—	—
* Hedophyllum nigripes* (BC)	Laminariales	MZ156046	38	25	10*^†^	52,071	—	—	—	—	—
* Hedophyllum subsessile*	Laminariales	MZ156062	38	25	4^†^	38,640	MZ156036	147	29	1	130,548
* Laminaria ephemera*	Laminariales	MZ156055	38	25	3	37,929	MZ156035	147	29	1	130,610
* Lessonia spicata*	Laminariales	MZ156056	38	25	3	37,097	MZ156034	147	28	1	130,301
* Lessonia variegata*	Laminariales	MZ156057	38	25	3	38,709	—	—	—	—	—
* Lessoniopsis littoralis*	Laminariales	MZ156066	38	25	4	38,444	MZ156033	147	29	1	130,839
* Macrocystis pyrifera*	Laminariales	—	—	—	—	—	MZ156032	147	29	1	130,196
* Pelagophycus porra*	Laminariales	MZ156058	38	25	3	37,465	—	—	—	—	—
* Pleurophycus gardneri*	Laminariales	MZ156059	38	25	4^†^	39,142	—	—	—	—	—
* Postelsia palmaeformis*	Laminariales	MZ156060	38	25	3	37,455	MZ156031	147^j^	28	1	129,997
* Pterygophora californica*	Laminariales	MZ156061	38	25	3	38,715	MZ156029	147	29	1	130,581
* Thalassiophyllum clathrus*	Laminariales	MZ156067	38	25	3	37,643	—	—	—	—	—

Information on previously sequenced species is a subset of available data, selected on the basis of unique features. The “core” set of mitochondrial genes includes rRNA genes, while the core set of plastid genes includes rRNA and *ycf* genes; fragments of *rpl*21 and *ycf*37 typically occur on inverted repeat regions in the listed plastid genomes but are not counted in the totals (when full genes are captured on the repeat, this is denoted with subscripts). Only unique genes are counted (i.e., repeats counted once). In the mitochondrial genomes, occurrences of intron viral elements are counted as ORFs and are indicated by an *, while † indicates a putative (pseudo)gene of the T7-like RNA polymerase gene first reported in *Pylaiella littoralis* (Oudot-Le Secq et al. 2001). ^ indicates that only a partial genome was assembled. AK = Alaska (USA); BC = British Columbia (Canada).

a *nad*6 occurs as two smaller fragments or subunits.

b *rpl*21 occurs as two copies captured on inverted repeat regions.

c *rbc*R and *rpl*32 missing.

d *syf*B missing.

e *rpl*21, *psb*A, and *rpl*32 all occur as two copies captured on inverted repeat regions.

f Hypothetical protein *Escp*36 occurs as two copies captured on inverted repeat regions

g *rpl*32 missing.

h orf258 occurs as two copies captured on inverted repeat regions.

i *rpl*31 missing.

j *ycf*37 occurs as two copies captured on inverted repeat regions.

The core content of plastid and mitochondrial protein-coding genes (including rDNA loci) is highly consistent across lineages of brown algae, with only a small number of gene losses or gains evident in each case. Specifically, *syf*B is missing in the plastomes of Fucales, *rbc*R and *rpl*32 are missing in the only available Dictyotales plastome (*Dictyopteris divaricata*; Liu et al. 2017), *rpl*32 is missing in the plastome of *Pleurocladia lacustris* but not in other plastomes of the same order (Ectocarpales). Moreover, the only instance of apparent mitochondrial gene loss is *rpl*31 in *Analipus japonicus,* the only Ralfsiales mitochondrial genome sequenced to date. While the fate of these genes remains unclear, it is possible that some may have been transferred to the nucleus, as has frequently been observed in plastid genomes of other lineages (e.g., Martin et al. 2002) and Stramenopiles (Ruck et al. 2014). This possibility should be explored in more detail when more high-quality nuclear genomes become available for these lineages. We attempted to search the *Sargassum* genome (Wang et al. 2020) for *syf*B with both tBLASTn (Altschul et al. 1990) and Bowtie2 (Langmead and Salzberg 2012) (read mapping using 60% similarity threshold) and using every *syf*B sequence described in this study as query sequences. However, we failed to obtain a convincing (>50% similar sequence identity) match, indicating that this gene has been completely lost from at least this lineage of Fucales. While gain and loss of core organellar genes appear to be limited across brown algal orders, Bringloe *et al.* (2021) report on an organellar genome from a putatively parasitic brown alga featuring several genome rearrangements, gene duplications and losses (including photosynthesis-related genes), as expected in plastid genomes of heterotrophic plants (e.g., Graham et al. 2017).

We found that some previously reported cases of gene loss in brown algal plastomes appear to be the result of annotation errors. For example, *ycf*17 was identified as present in Fucales and *ycf*54 and *pet*L were found in Laminariales and Chordales contrary to the interpretation of Graf et al. (2017). Moreover, the putative pseudogenization of *ycf*37 in *Laminaria solidungula* (Laminariales) reported by Rana et al. (2019) appears to be the result of incorrectly interpreting the fragmented portion of this gene that occurs in one of the inverted repeat regions (while the intact gene straddles the other inverted repeat region). It is worth noting that tRNA gene gain and loss appear to be more common than in other genes in both organelles (table 1). However, we did not formally analyze the specific events here. Altogether, our results, along with other investigations of organellar gene content (Graf et al. 2017), indicate that the core gene sets of both organelles were likely established early in the evolution of brown algae. This comes as no surprise; mitochondrial gene sets are hypothesized to have become established early on in eukaryotic evolution when most of the endosymbiont genes were lost or transferred to the host nucleus (Adams and Palmer 2003). We note, however, that our results bring the totals to 9 and 7 orders sequenced for mitochondrial and plastid genomes (respectively) out of 19: additional sequencing is required to fully understanding organellar genome gene loss across the brown algal phylogeny. Nevertheless, given that sequenced organellar genomes are from diverse orders spread across the brown algal phylogeny, the patterns reported here are likely broadly representative of the entire group.

The presence of conserved ORFs in some taxa may point to novel gene gain in specific lineages of brown algae. Three mitochondrial ORFs are highly conserved across taxa sampled here, and one plastid ORF (in addition to previously reported *ycf* genes) is highly conserved, although this ORF appears to be absent in *Chorda asiatica* (table 1). Novel lineage-specific ORFs appear to be particularly prevalent in Ectocarpales and have been reported previously (Oudot-Le Secq et al. 2001; Le Corguillé et al. 2009). Moreover, numerous ORFs were identified in *Pseudochorda nagaii* and *C. asiatica* (Chordales); however, none appear to be homologous (alignable) between the two species. These ORFs are of unknown origin but could be the consequence of either horizontal gene transfer (e.g., Secq et al. 2002; Ikuta et al. 2008) or de novo gene formation (see Oss and Carvunis 2019 for a review). We further report several novel ORFs occurring in introns (predominantly in LSU and *cox*I) that resemble T7-like RNA polymerase genes or pseudogenes (in *Desmarestia aculeata, Hedophyllum nigripes, Hedophyllum subsessile, Pleurophycus gardneri*, and *Ps. nagaii*). These are similar to those first reported in *Pylaiella* (Ectocarpales; Oudot-Le Secq et al. 2001). Two of the intact ORFs in *Ps. nagaii* flank one of these T7-like RNA polymerase pseudogenes which may provide further evidence of lateral gene transfer. We discuss these introns in more detail below.

### Sequence Divergence is Greater across Mitochondria than Plastids

**Fig. 1.**
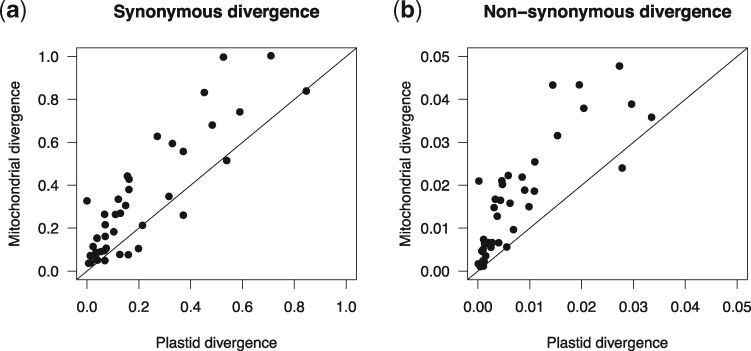
Plastid and mitochondrial sequence divergence among select brown algal taxa. Correlations of synonymous (a) and nonsynonymous (b) divergence between plastid and mitochondrial protein-coding regions are shown, with each data point representing a branch of the phylogeny containing the subset of species used in the analysis (*n* = 23). Divergence values were estimated using concatenated alignments of 124 plastid genes and 34 mitochondrial genes. Solid lines indicate a ratio of 1:1 between plastid and mitochondrial divergence.

**Fig. 2.**
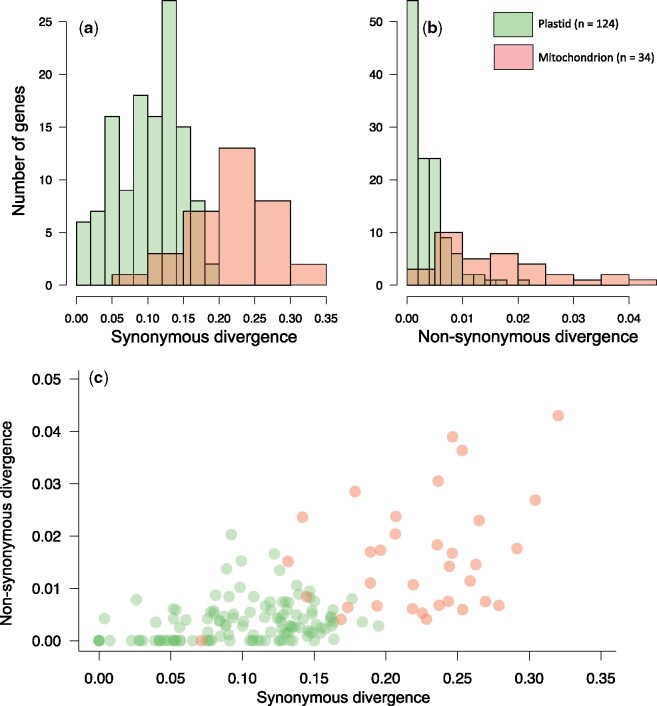
Variation in sequence divergence across organellar protein-coding genes. Histograms show the frequency of median synonymous (a) and nonsynonymous (b) divergence for each individual protein-coding gene across the 23-taxon brown algal phylogeny. (c) Correlation between nonsynonymous and synonymous divergence. In all panels, red represents mitochondrial genes, while green represents plastid genes.

We used synonymous and nonsynonymous divergence to compare the rates of sequence evolution across genes and organelles. Synonymous divergence is generally considered to be a proxy for neutral substitution rates, while nonsynonymous divergence is usually lower due to purifying selection; the ratio of these two (dN/dS) can then be used to infer the strength of purifying selection. Between *Dictyopteris* (the most evolutionary distinct species that we considered; order Dictyotales) and the clade containing all other brown algal species, synonymous divergence was highly saturated in both the plastid and mitochondrion (dS > 1). Thus, in order to allow for meaningful comparison of divergence between organellar genomes, we excluded *Dictyopteris* from analyses of molecular divergence. In all cases, nonsynonymous divergence was not saturated (dN ≪ 1). We first compared synonymous and nonsynonymous divergence across entire organelles. We found that across almost all branches of brown algal phylogeny, both synonymous and nonsynonymous divergence rates are several-fold greater across mitochondrial genes than plastid genes (fig. 1). Next, we compared median synonymous and nonsynonymous divergence rates of each gene from both organelles (fig. 2). This further corroborated that mitochondrial genes evolve at faster rates than plastid genes, but also document overlaps in rates between loci across the two organelles. Where they overlap, the gene-wise relationships (i.e., slopes) between synonymous and nonsynonymous rates are similar (fig. 2*c*). The higher nonsynonymous divergence in mitochondrial genes relative to plastid genes presumably reflects underlying differences in mutation rate between organelles. However, the variation around the relationship between synonymous and nonsynonymous divergence may also reflect differences in the strength of selection acting on different genes.

**Fig. 3.**
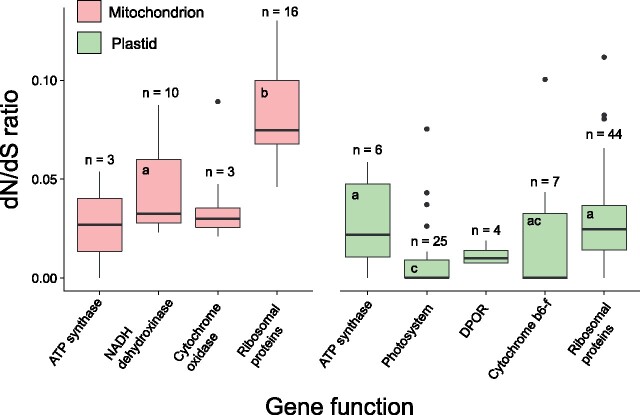
dN/dS across major organellar gene classes. Boxplots show the median dN/dS for classes of mitochondrial and plastid genes across the 23-taxon brown algal phylogeny. Letters indicate significant differences between dN/dS ratio across major gene classes. Classes with less than five genes were not included in statistical analysis.

Analysis of dN/dS ratios reveals that genes from all major organellar classes are under strong purifying selection: median dN/dS ratios for all gene classes investigated were ≪ 1 (fig. 3, supplementary figs. 1 and 2, Supplementary Material online). This provides strong evidence of purifying selection acting on these protein-coding genes (Yang et al. 2000). Despite this, the dN/dS ratios do vary significantly across the major gene classes (ANOVA: F = 15.18; *P *<* *0.001; df = 5, 102). Consistent with past work for other taxonomic groups (e.g., Guisinger et al. 2008), photosystem genes had particularly low dN/dS ratios, suggesting that they experience the strongest purifying selection. However, there is also clear variation within this gene class, indicating that not all photosystem genes are under equally strong selection. For example, while most photosystem II genes had dN/dS values below 0.001, *psbK* and *psbV* had values of 0.075 and 0.026, respectively. In contrast to photosystem genes, ribosomal genes appeared to be under somewhat weaker purifying selection (though dN/dS still ≪1), with significantly greater dN/dS values inferred for mitochondrial genes than their plastid counterparts (post hoc comparison: *P *<* *0.001). ATP synthase genes were somewhat intermediate between these two other gene classes. However, in contrast to ribosomal genes, ATP genes had similar dN/dS values from both organelles, pointing to an interaction between gene function and organelle type, and making broad generalizations about variation in purifying selection challenging.

Overall, our findings are consistent with previous work on other taxa with secondary plastids (Smith 2015, Smith and Keeling 2015), concerning the finding that mitochondrial mutation rates are higher than plastid mutation rates in these taxa. This general pattern contrasts with that observed in taxa with primary plastids. In green algae and land plants, DNA sequence evolution in the mitochondria is much slower than in plastids, except for within the inverted repeat region (Clegg et al. 1994, Smith 2015, Smith and Keeling 2015). While the drivers of variation in mutation rates are not fully understood, they likely reflect differences in the efficiency of DNA replication machinery between organelles and also across taxonomic lineages (Eyre-Walker and Gaut 1997; Drouin et al. 2008; Zhu et al. 2014; Smith 2015; Gualberto and Newton 2017). Endosymbiotic history (i.e., number of endosymbiosis events) may also play an important but poorly understood role in this regard (Smith and Keeling 2015).

To aid with the future selection of organellar genes for phylogenetic analysis and DNA barcoding or metabarcoding, as has recently been done for red algae (Janouškovec et al. 2013; Graf et al. 2017; Zhan et al. 2020), we provide a supplementary table S1, Supplementary Material online with median and interquartile range (IQR) of both synonymous and nonsynonymous divergence (supplementary table S1, Supplementary Material online). While the median gives a general measure of sequence evolution rate, IQR gives a measure of differential rate across taxa. Knowledge of these two properties for each gene should help to make selection of genetic markers more targeted towards the desired phylogenetic resolution (i.e., sequence rate) in future studies that focus on subsets of genes (Janouškovec et al. 2013). We note that our results are similar qualitatively to the results of Graf et al. (2017).

### Plastids Are More Structurally Variable than Mitochondria

**Fig. 4.**
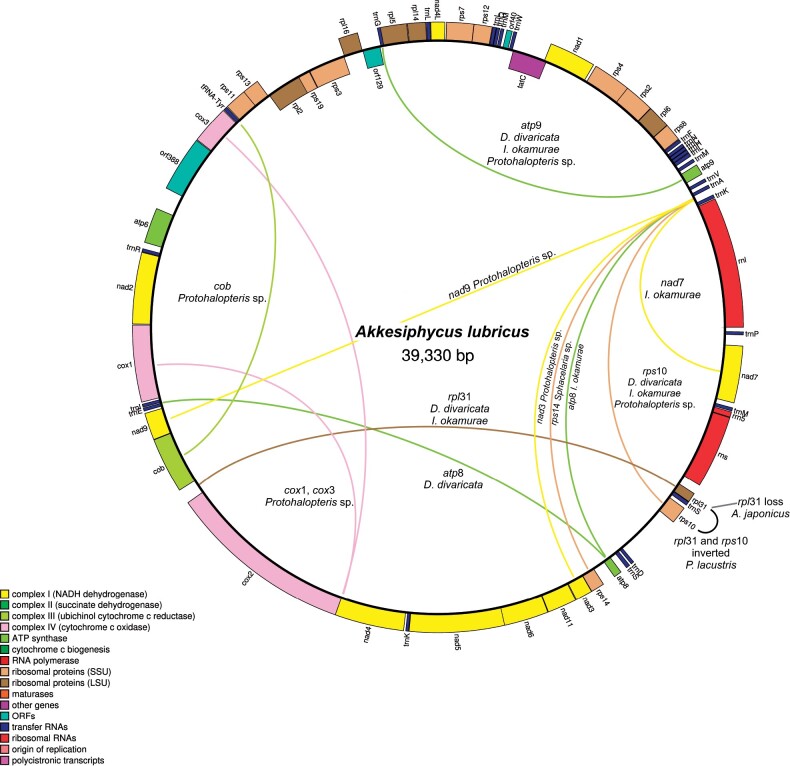
Mitochondrial genome structure of *Akkesiphycus lubricus* (Chordales), an arrangement widely conserved in brown macroalgae. Minor rearrangements (including inversions) are depicted for *Analipus japonicus* (Ralfsiales), *Dictyopteris divaricata* (Dictyotales), *Ishege okamurae* (Ishegeales), and *Pleurocladia lacustris* (Ectocarpales). More substantive rearrangements are indicated between *Protohalopteris* and all other brown algae based on a partial mitochondrial genome assembly of the former.

Mitochondrial genomes were almost entirely colinear across species examined here, with mostly minor rearrangements between taxa (fig. 4). The exception here was the partial mitochondrial genome of *Protohalopteris* sp. (Sphacelariales) which has substantial variation in gene order relative to all other examined taxa (fig. 4). All other taxa share a fairly conserved gene order, with the few observed rearrangements of individual genes appearing to flank the rRNA genes (fig. 4).

**Fig. 5.**
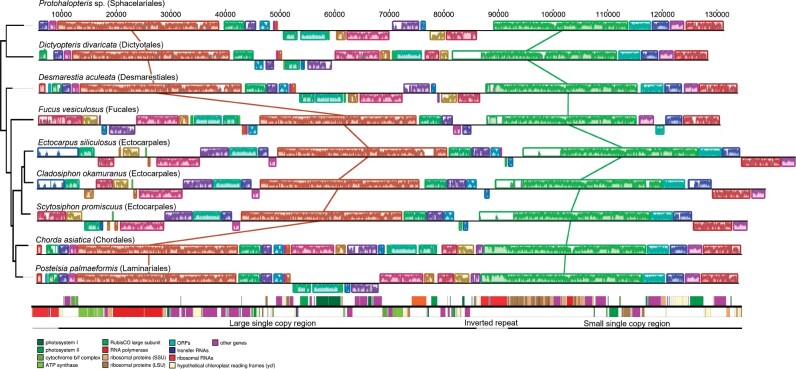
Structural evolution in plastid genomes across orders of brown macroalgae. The gene map across the bottom shows the arrangement for *Postelsia palmaeformis*. Only one representative species with a particular genome structure is shown. The maximum likelihood tree on the left was constructed using *rbc*L, and is consistent with known phylogenetic relationships between taxa (Bringloe et al. 2020).

In contrast, plastids varied substantially in gene order (fig. 5). Across different orders of brown algae, plastome rearrangements occurred in both the large (LSC) and small single copy sections of the plastome. Infraorder variation in plastid architecture was only present in Ectocarpales, which have been described previously as variable (Graf et al. 2017; Choi et al. 2020), and in Chordales. In the latter, *Akkesiphycus lubricus* and *Ps. nagaii* were found to have identical plastome architecture to Laminariales. However, *C. asiatica* possesses a unique LSC organization relative to other members of the Chordales–Laminariales clade that is presumably the result of a single inversion event. This type of rearrangement (mid-single copy inversion) is consistent with the types of rearrangements generally seen between orders or between members of Ectocarpales (fig. 5).

Variation across organellar lineages in both sequence evolution and genomic rearrangements is believed to arise through differences in DNA repair mechanisms and efficiency (e.g., Zhu et al. 2014; Smith 2015; Smith and Keeling 2015; Gualberto and Newton 2017). Patterns of organellar sequence and structural evolution are consistent with the hypothesis that coding and noncoding regions are treated differently in terms of DNA repair (Davila et al. 2011) and may vary independently across lineages (Smith and Keeling 2015). Plastids here showed slower rates of evolution in the coding regions (even “silent,” synonymous mutations) but far greater variation in structure. It is possible that these patterns both reflect efficient repair pathways that correct mutation through gene conversion, ultimately leading to higher structural variation but more conserved gene sequences (Gualberto and Newton 2017). In brown algae, with the exception of *Protohalopteris* sp. (Sphacelariales), rearrangements in the plastid have generally occurred along noncoding regions, preserving gene integrity. Moreover, we hypothesize that these rearrangements have preserved coordinately transcribed polycistronic units; perhaps explaining why many inferred plastome inversions have included long stretches of DNA (fig. 5). These patterns are consistent with the notion that sequence evolution rates in coding regions can be independent of evolution at the noncoding regions, an emerging pattern across organellar genomes (Smith and Keeling 2015). In addition to variation in the efficiency of DNA replication machinery, variation in plastid architecture may arise through processes involving the inverted repeat (IR) region. For example, the edges of the IR region can gradually expand and contract, and may also be susceptible to bordering inversions associated with the flip-flop recombination that affects the IR (Palmer 1983; Wicke et al. 2011; Wang and Lanfear 2019).

Recent work has suggested that variation in plastid architecture may be linked to reproductive strategy and mode of organellar inheritance (Crosby and Smith 2012; Choi et al. 2020). Our results may provide some additional support for this hypothesis. Biparentally inherited plastomes from Ectocarpales (which have isogamous reproduction) are structurally more variable than those of Chordales, Laminariales, or Fucales, which generally have oogamous reproduction and are thus, presumably, maternally inherited plastids (Choi et al. 2020). Moreover, the most structurally novel plastome (and the largest) sequenced in this study is from Sphacelariales, an order with a diversity of reproductive modes and strategies. *Protohalopteris* sp., in particular, tends to reproduce either asexually or through isogamous or anisogamous reproduction (Gibson 2013). In contrast, we detected virtually no variation in plastome architecture across the entire Laminariales order, suggesting a high degree of conservatism despite an increasing number of replicate genomes. Nonetheless, this hypothesized association is challenging to assess rigorously, at present, because of limited phylogenetic replication, and differences in the timing of diversification of different taxonomic orders (i.e., Ectocarpales diverged before Laminariales Silberfeld et al. 2010).

### Conservation, Loss, and Lateral Gene Transfer of Introns

We identified two distinct types of introns found mostly in the same genes across brown algae. We found group I introns in the tRNA-Leu-UAA of all newly sequenced plastomes. This intron has been previously documented in other brown algal taxa (e.g., *Fucus vesiculosus*, *Costaria costata*; Kuhsel et al. 1990, Le Corguillé et al. 2009) but has been found inconsistently both within and across species. For example, it has been found in some (but not all) kelps (Laminariales) and is absent from species in Ectocarpales sequenced to date. We reanalyzed previously published plastomes using the latest version of tRNA-scan (data not shown) and found that the presumed absence of the tRNA-Leu intron in some kelp plastomes is actually an artifact of the tRNA detection algorithm used. While this intron is clearly present when using the “organelle” or “general tRNA” algorithm in tRNA-scan or in Aragorn (Laslett and Canback 2004), it is missed by the “bacterial” algorithm in tRNA-scan. However, we note that the organelle algorithm for tRNA detection was not available in tRNA scan until its latest version, released in 2019. Future detection of tRNAs during the annotation of brown algal genomes should therefore be done with care to use the appropriate tRNA detection algorithm and to ensure consistency when inferring evolutionary loss and/or gain of tRNAs. The only plastid genome that we reanalyzed that did not have a tRNA-Leu-UAA group I intron was *E. siliculosus*, which was originally analyzed using Aragorn and yielded this same result (Corguillé et al. 2009); thus, this appears to be a genuine intron absence in this species.

**Fig. 6.**
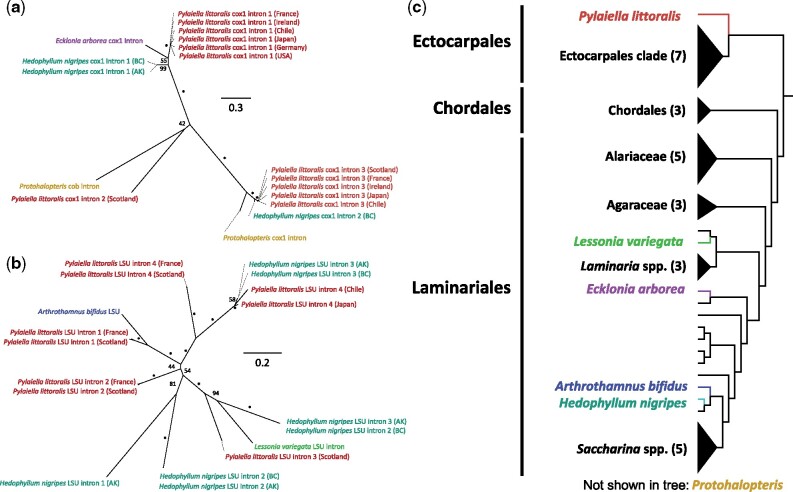
The distribution of introns across brown algal mitochondrial genomes. Panels a, b show maximum likelihood (ML) phylogenetic reconstructions (unrooted) of group II introns found in the mitochondria of brown algae. Panel a includes introns found in *cob* and *cox1*; panel b includes introns from the mitochondrial LSU region. Asterisks (*) indicate bootstrap support values > 97%, while most other support values > 50% are shown (several omitted among very closely related sequences). Panel c shows a cladogram of the Ectocarpales-Chordales-Laminariales clade including only species for which the mitochondrial genome has been sequenced (based on Starko et al. 2019, Bringloe et al. 2020). *Protohalopteris* (Sphacelariales) is a member of a distantly related brown algal lineage and is not included in the cladogram. No other sequenced brown algal mitochondria possess homologous introns.

In addition to our consistent detection of the previously reported group I intron, we found that mitochondrial group II introns (both groups IIA and IIB), which have been documented previously only from the Ectocarpales species *P. littoralis* (Ikuta et al. 2008), are widespread across taxa in that order. Group II introns in Stramenopiles contain degraded ORFs that code for retrotransposons (Ikuta et al. 2008). Thus, they are believed to be selfish genetic elements (Bonen and Vogel 2001). Here, we found that the mitomes of both sequenced individuals of *H. nigripes* are drastically inflated, ranging from 52,041 to 52,072 bp, similar in size to *P. littoralis* (Ikuta et al. 2008). This reflects the presence of several introns that account for more than 90% of the ∼20 kb size difference between the mitogenomes of *H. nigripes* and the closely related species *H. subsessile*. Mitochondrial genome size inflation driven by proliferation of introns has been seen in other algal lineages, and could reflect any of a number of factors that limit purifying selection (e.g., reduction in effective population size; Repetti et al. 2020). We also found group II introns in the mitomes of the Laminariales species *Arthrothamnus bifidus*, *Lessonia variegata*, and *Eisenia arborea*, but not in the mitogenomes of close relatives of these taxa. This demonstrates that group II introns are widespread across the Laminariales but have likely been either gained or lost on several occasions throughout the diversification of kelp mitochondrial genomes. We also found three introns in the partial mitochondrial genome assembly of *Protohalopteris* sp. (Sphacelariales): two introns in *cox*1 and *cob* that share high similarity with group IIA introns from Laminariales and *Pylaiella* (Ectocarpales), and one additional group II intron in *cox*2 that was most similar to the group II intron found in *cox*2 from *Ulva pertusa* Kjellman (Liu et al. 2017) a distantly related green alga. The low identity between the latter two introns suggests a lateral gene transfer event. In all other instances in which we detected mitogenome introns, group IIA introns were found in the *cox*1 gene and group IIB introns were found in the LSU rRNA gene (fig. 6), including previously published group II introns from *P. littoralis* (Ikuta et al. 2008).

We constructed phylogenies of each group of introns and confirmed that these introns have a complex evolutionary history that is likely indicative of widespread lateral gene transfer and multiple recurrent invasions. While some clades of introns are restricted to individual species, there is widespread incongruency between intron phylogenies (fig. 6*a* and *b*) and the known evolutionary relationships between eukaryotic hosts (Silberfeld et al. 2010; Starko et al. 2019; Bringloe et al. 2020) (fig. 6*c*). For example, there is a strongly supported cluster containing all *cox*1 intron 1 sequences (fig. 6*a*) from *P. littoralis* (Ectocarpales), *Ei. arborea* (Laminariales), and *H. nigripes* (Laminariales). However, both *P. littoralis* and *H. nigripes* have several other introns that are not part of this cluster. Moreover, close relatives of each of these taxa do not possess this (or other) introns (fig. 6*c*). Ikuta et al. (2008) previously hypothesized that group II introns have recurrently and recently invaded *P. littoralis*. Our results support the hypothesis of multiple intron invasions because the phylogeny of known group II introns across the brown algae (fig. 6*a* and *b*) is highly incongruent with a hypothesis of vertical inheritance alone (the tree in fig. 6*c*). If these introns were present in the most recent common ancestor of the Ectocarpales–Chordales–Laminariales clade and were subsequently lost in many taxa, this would have required that these introns were retained across laminarialean phylogeny until recently and then were systematically purged from all but a few species and genes. The latter hypothesis is difficult to justify mechanistically and would fail to explain the strong conflict between intron and species phylogenies.

Due to the observed close similarity between mitochondrial introns from distantly related species, we postulate that lateral gene transfer (or invasion) of group II introns could have either occurred through direct contact between these taxa or through a vector, such as a microbe or virus that tightly associates with a wide range of brown algae. Given that *A. bifidus*, *Lessonia variegata*, and *Ei. arborea* do not co-occur and *H. nigripes* currently only narrowly overlaps with *Ei. arborea* in British Columbia, direct lateral gene transfer events could likely only have occurred between *P. littoralis* and the four laminarialean kelps, and likely not from kelp to kelp. *Pylaiella littoralis* is a known epiphyte of large brown algae (including Laminariales), a life history trait that could clearly allow for the contact required for lateral transfer of *P. littoralis* introns to other species (as observed in land plants Rice et al. 2013). *Pylaiella littoralis* can also be epiphytic on fucoids, especially in the Atlantic. The few mitomes available for fucoids do not possess group II introns; however, future sequencing efforts may reveal group II mitochondrial introns in Fucales if the spread of these sequences is linked to epiphytism. An alternative hypothesis is that these introns are spread by microbes or viruses and represent multiple infections (Ikuta et al. 2008) that may be removed from genomes at different rates across taxa. Brown algae have a wide range of symbionts (Kohlmeyer and Demoulin 1981; Balakirev et al. 2012; Weigel and Pfister 2019; Lemay et al. 2021), parasites (Kohlmeyer and Demoulin 1981; Müller et al. 1999; Bringloe et al. 2021), and specialist viruses—including some with very large genomes (Müller et al. 1998, Delaroque et al. 1999)—that could act as vectors of group II introns, spreading them across brown algal taxa (Ikuta et al. 2008). Group II introns have also been found inconsistently in the *cox*1 and *rnl* genes of diatom mitomes (Pogoda et al. 2019), suggesting that Stramenopiles may be sensitive to intron invasion at these particular loci.

## Conclusions

In sum, we sequenced a large number of organellar genomes from brown algae, substantially expanding the number of reference genomes available for this group. We leveraged this newly available genomic data to compare sequence and genome structural evolution across brown algal organelles. We confirmed that a core set of mitochondrial and plastid genes are conserved across the brown algae, with few cases of gene gain/loss in the orders sequenced thus far. We found that mitochondrial genes generally evolve at a faster rate than plastid genes, with few exceptions. However, structural rearrangements (especially inversions) are more prevalent across plastid genomes than mitochondrial genomes. Finally, we demonstrated that group II introns are widespread but inconsistently found across mitochondrial genomes of different lineages of Laminariales. This likely reflects lateral gene transfer of introns between species or from a vector that is associated with a range of brown algae, given the observed incongruence between host and intron phylogenies and presence of closely related introns in different genes of the same species. We postulate that epiphytism, symbiosis, or parasitism may facilitate this intron transfer process. Overall, our findings substantially improve our understanding of the patterns of organellar genome evolution in this ecologically and economically important clade of photoautotrophic algae.

## Materials and Methods

### Sample Acquisition, Extraction, and Sequencing

Samples were collected from various locations worldwide, with a particular focus on the North Pacific, where laminarialean diversity is greatest (table 1). Most were dried in silica gel, but three of the samples (*Ak. lubricus*, *Ps. nagaii*, and *C. asiatica*) were taken from cultures housed in the Kobe University Macroalgal Culture Collection in Hokkaido, Japan. DNA extraction and sequencing were done as part of a large phylogenomics project (Starko et al. 2019). DNAs were extracted using one of three methods: 1) Qiagen DNeasy Plant Extraction Mini Kit as per manufacturer instructions, 2) brown algal extraction buffer (Saunders and McDevit 2012) followed by the Qiagen Wizard DNA Clean-Up Kit (Qiagen, Hilden, Germany), or most often 3) a modified CTAB protocol (Doyle and Doyle 1987, Rai et al. 2003). Details on sample-specific methods are reported in Starko et al. (2019); one exception was the *H. nigripes* sample from Alaska which was not included in that study and which was extracted using the CTAB method here. Library preparation and quality control methods follow Starko et al. (2019).

### Assembly and Annotation

While genome-scale organellar gene sets for most samples were used in Starko et al. (2019), here we assembled these sequences into full plastid and mitochondrial genomes. Genomes were primarily assembled using NOVOPlasty 2.7.2 (Dierckxsens et al. 2017) which was developed to assemble full organellar genomes directly from raw next-generation sequencing data. We used mostly default settings, which are optimized for samples with complete genome coverage. However, for lower coverage samples, we reduced k-mer size from 39 to 23 as per recommendations from the developers of NOVOPlasty (Dierckxsens et al. 2017). We assembled the plastome of *Egregia menziesii*, which was sequenced using earlier shorter read technology (Starko et al. 2019), using de novo assembly in CLC Genomic Workbench v 6.5.1. We selected contigs that were at least 500 bp in length and >10× coverage. We bridged gaps between several contigs and confirmed contig overlap using Sanger DNA sequencing. We designed custom primers for amplification and Sanger sequencing using Primer3 (Koressaar and Remm 2007), performing amplifications using Phusion High-Fidelity DNA Polymerase (Thermo Fisher Scientific, USA) and sequencing using BigDye Terminator v.3.1 (Applied Biosystems, Inc. Foster City, USA). We performed amplification following the general methodology in Graham and Olmstead (2000), with modifications.

Genomes were annotated using GeSeq (Tillich et al. 2017) using *Co. costata* and *E. siliculosus* as reference genomes for plastome annotation, and *Co. costata*, *Desmarestia viridis*, and *E. siliculosus* as reference genomes for mitome annotation. The program MFannot (https://megasun.bch.umontreal.ca/cgi-bin/dev_mfa/mfannotInterface.pl) was used to further confirm the GeSeq annotations and identify novel open reading frames (ORFs). Annotations were manually checked and corrected using Geneious Prime (Kearse et al. 2012). We annotated tRNA genes using tRNA-scan v2.0.5 (Chan and Lowe 2019), specifying the “other mitochondrial” algorithm for mitomes and the “organellar tRNA model” for plastomes.

### Sequence Divergence Analysis, Whole-Genome Alignments, and Intron Detection

We estimated nonsynonymous and synonymous divergence of protein-coding genes, focusing on 33 mitochondrial genes and 123 plastid genes. We considered 23 species of brown algae for which both organelles have been sequenced (Chordales: *Ak. lubricus, C. asiatica, Ps. nagaii*; Fucales: *Coccophora langsdorfii, F. vesiculosus, Sargassum horneri, Sargassum thunbergii*; Desmarestiales: *Desmarestia* spp.; Ectocarpales: *E. siliculosus, Endarachne binghamae;* Laminariales: *Alaria marginata, A. bifidus, Ei. arborea, Ecklonia radicosa, H. subsessile, Laminaria ephemera, Lessonia spicata, L. variegata, Macrocystis pyrifera, Postelsia palmaeformis, Saccharina japonica, Undaria pinnatifida*). *Desmarestia* spp. was incorporated into the analysis by using the published mitome of *D. viridis* and the plastome of *D. aculeata*, newly sequenced here. We aligned translated (amino acid) sequences using ClustalW2 (Larkin et al. 2007), checked them manually using AliView (Larsson 2014) and then reverse-translated amino acid alignments to codon-based alignments using Pal2Nal (Suyama et al. 2006). Regions with gaps and poorly aligned regions were removed using Gblocks (Castresana 2000) (using default parameters). Synonymous and nonsynonymous divergence was then estimated both for individual genes and for concatenated alignments using the maximum likelihood method implemented in the codeml software in PAML (Yang 1997), using a tree topology adapted from recent phylogenetic studies (Silberfeld et al. 2010; Liu et al. 2019; Starko et al. 2019) by pruning out taxa not included here. The tree file used as input is available as a supplementary file, Supplementary Material online. We extracted the median nonsynonymous (dN) and synonymous (dS) divergence and their ratio (dN/dS) from codeml outputs to make inferences about mutation rate and purifying selection within and across genomes. To test whether the degree of purifying selection varies by function, we conducted a rank-transformed ANOVA on median dN/dS values for genes from five plastid (ATP synthase, DPOR, Cytochrome b–f, photosystem, ribosomal) and two mitochondrial (NADH dehydrogenase, ribosomal) gene classes followed by a Tukey post hoc test implemented in R (R Development Core Team 2008). To test for divergence from colinearity of organellar genomes, we aligned whole organellar genomes using default settings in Mauve (Darling et al. 2004).

We initially detected introns using the built-in algorithm included in GeSeq (Tillich et al. 2017). These were further confirmed in the MFannot annotations, and the presence of reverse transcriptases was validated using InterProScan (Jones et al. 2014). We produced two intron alignments, one for all group IIA introns and the other for group IIB introns. We then conducted phylogenetic reconstruction of the intron sequences in RAxML (Stamatakis 2014) using a GTRGAMMA model of sequence evolution. Branch support was inferred using 1,000 bootstrap replicates.

### Data Availability

All raw sequence data are available on the NCBI Sequence Read Archive (SRA) as part of BioProject PRJNA530337. All organellar genomes are available on Genbank (see table 1).

## Supplementary Material

Supplementary data are available at *Genome Biology and Evolution* online.

## Acknowledgments

We respectfully acknowledge the Huu-ay-aht, Musqueam, Squamish, Tsleil-waututh, Songhees, Esquimalt and WSÁNEĆ Nations on whose traditional territory this research was conducted and whose lands many of these samples were collected. We thank K. Demes, M. Edwards, H. Kawai, E. Macaya, W. Nelson, N. Yotsukura & M. Lemay for contributing brown algal material. Thanks to staff at Bamfield Marine Sciences Centre and Hakai Institute (Calvert Island) for facilitating collection of some samples. We also thank P. Keeling for contributing to sequencing efforts and for helpful discussions regarding these findings. Funding for this project was provided by Natural Sciences and Engineering Research Council of Canada (NSERC) Discovery Grants to SWG and PTM. Funding was also provided by a Phycological Society of America Grant-in-Aid of Research Award to SS.

## Supplementary Material

evab124_Supplementary_Data
