# Supplementary material for: Genomic Rearrangements and Sequence Evolution across Brown Algal Organelles
Source: Genome Biol Evol. 2021 Jun 1;13(7):evab124. doi: 10.1093/gbe/evab124 (PMC8290108; doi:10.1093/gbe/evab124)
Supplement: evab124_Supplementary_Data [file evab124_supplementary_data.zip › Supplementary Figure and Table Captions_210109.docx]

**Supplementary Figure and Table Captions**

**Fig S1. dN/dS for mitochondrial genes (n = 34) estimated from representatives across the brown algae.** Shown is the median ratio of non-synonymous and synonymous sequence divergence for 23 species of brown algae.

**Fig S2. dN/dS for plastid genes (n = 123) estimated from representatives across the brown algae.** Shown is the ratio of non-synonymous and synonymous sequence divergence for 23 species of brown algae. Note the difference in scale between top and bottom panel.

**Table S1. Sequence divergence parameters estimated from representatives across the brown algae.** This table shows mean and median synonymous and non-synonymous sequence divergence for 23 species of brown algae. Also shown are the interquartile ranges of both synonymous and non-synonymous sequence divergence. Parameters are presented for 123 protein-coding genes from the plastid and 34 protein-coding genes from the mitochondrion.
