# Supplementary figures and images for: Genomic Rearrangements and Sequence Evolution across Brown Algal Organelles
Source: Genome Biol Evol. 2021 Jun 1;13(7):evab124. doi: 10.1093/gbe/evab124 (PMC8290108; doi:10.1093/gbe/evab124)

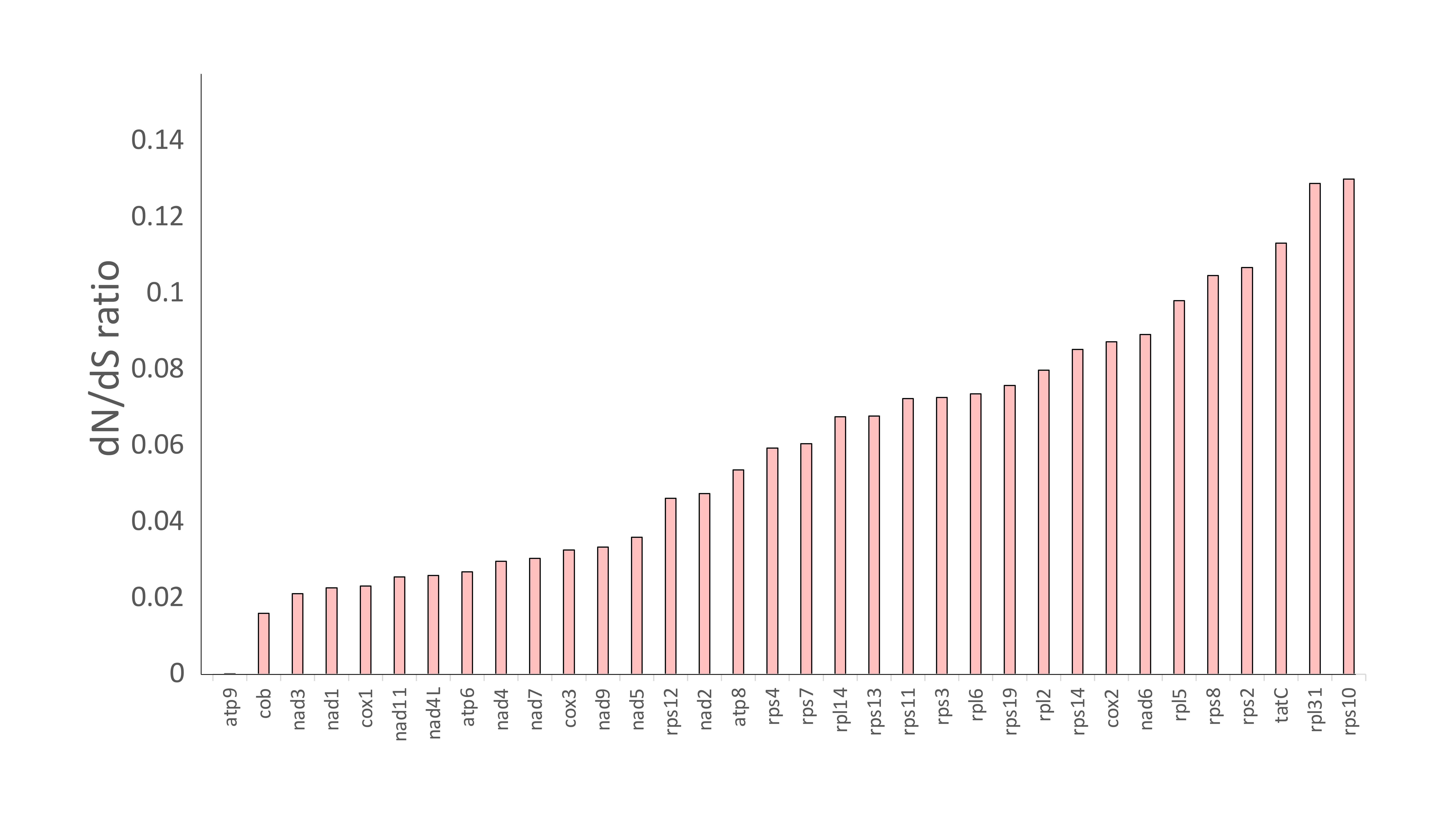

Supplement: evab124_Supplementary_Data [file evab124_supplementary_data.zip › FigS1.tif]

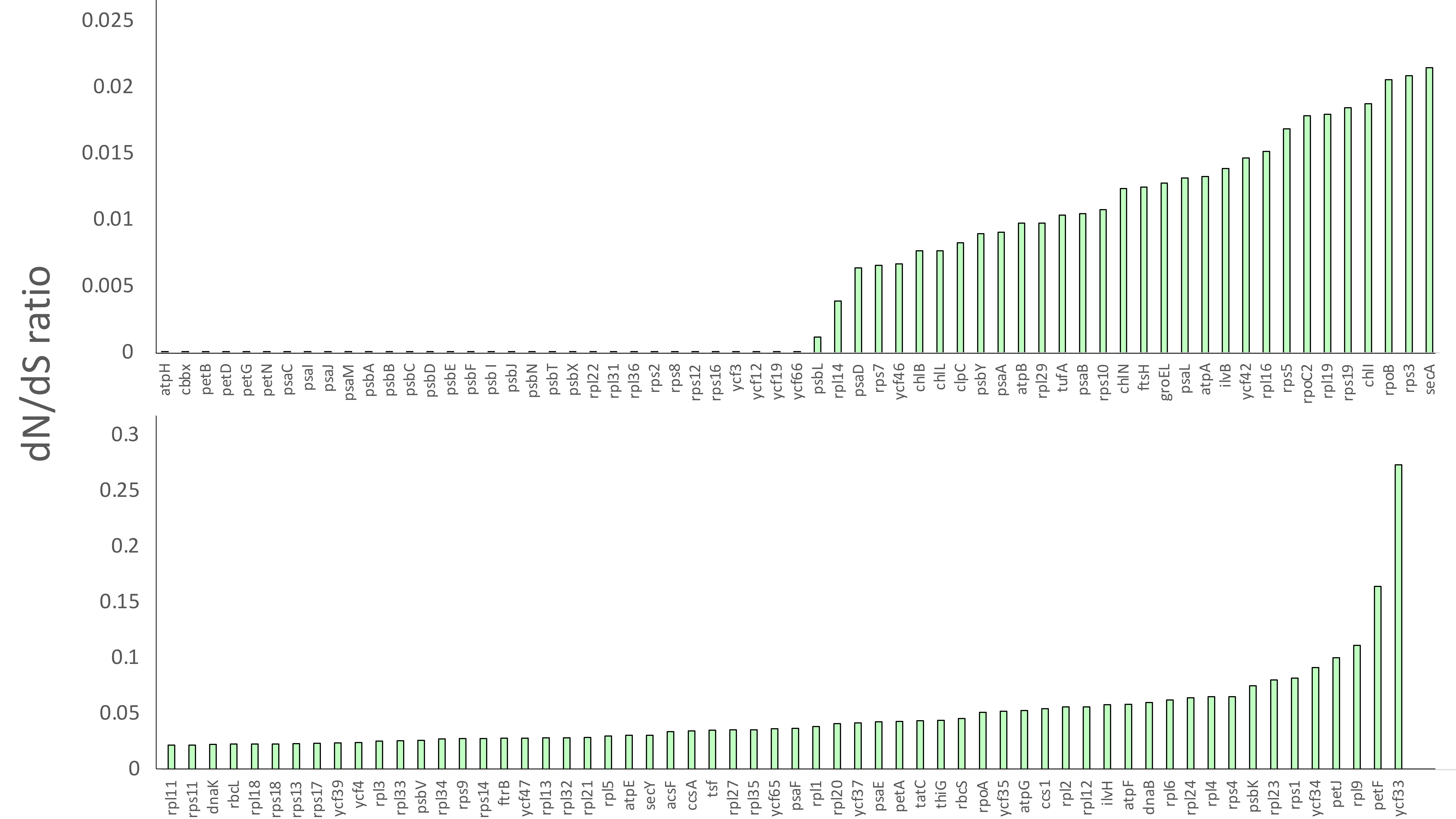

Supplement: evab124_Supplementary_Data [file evab124_supplementary_data.zip › FigS2.tif]
